# Round the Hospitals

**Published:** 1918-12-21

**Authors:** 


### ROUND THE HOSPITALS.

At a special service held in the Assembly Hall at Belfast on December 1, numerous detachments of the British Red Cross and St. John of Jerusalem attended, and there was a crowded congregation. The special preacher was Dr. Smythe, who has lately returned from France, and in the course of his sermon he told his hearers that during the time he spent at the Front he had been able to gain a knowledge of the work carried on by "the angels dressed in women's clothes."

---

No date has yet been fixed for the opening of the Peace Conference, but Mr. Lloyd George and Mr. Bonar Law will spend Christmas in France, and some 400 officials are going to Paris for duty in various directions on the staff. The headquarters of the British delegates will be the Hôtel Majestic. Among the large number of women who form part of the staff are many from the Foreign Office and Board of Trade. A British doctor and a certain number of nurses have also received orders to accompany the delegates so that in the event of illness efficient arrangements can be made. This is a very wise precaution, in view of the prevailing epidemic, and the shortage of nurses in the French capital.

---

Under Lady Rhondda as President a woman's council has been formed to safeguard the interests of women in regard to the Ministry of Health. The movement is in its initial stages, but the Council is disposed to press for the establishment of the Health Ministry; for adequate female representation in every department of it, and for the formation in connection with it of an advisory council of women constituted on a democratic basis. It is quite true that working women are not consulted about the numerous schemes which well-intentioned people frame for their benefit, and that if they could be helped to think and to devise measures for the improvement of their homes and the health of their children much new light might be thrown upon puzzling problems. We trust Lady Rhondda will be able to get into touch with the right people in forming her council.

---

That military hospitals for disabled men are to be kept open for six months is a pronouncement which gives general satisfaction. The little hospitals wait in vain to learn their fate. The worst method to adopt in regard to them is that of steadily marking out their patients while never replacing them, until the hospital slowly expires of inanition. It ought to be recognised that this slow decease is very trying to the staff, who can make no other plans while the hospital has still to be kept in working order, which is very costly.

---

Almost every class of worker is being granted extra leave this "Victory" Christmas season, but so far we have seen no mention of holidays to nurses. It is not very practicable to lessen the staff at this festival which is traditionally and quite rightly an occasion when the first consideration is to make the patients happy by united effort. But now that the influenza epidemic is passing it woul[illegible] be not merely graceful, but just, for managers to give an extra week or ten days' leave in January to all members of the staff in turn in grateful recogn[illegible]tion of the many hours of over-time cheerfully sur[illegible]rendered during times of stress. Most of all d[illegible] the matrons and heads of departments need relaxa[illegible]tion and refreshment of spirit after the heavy season. To make adequate arrangements for their relief would in many cases ward off a painful period of lassitude, after great exertions. To be compelled to "worry through" without pause under a sense of brain fatigue may pave the way to subsequen[illegible] trouble.

---

We are glad to learn that a scholarship schem[illegible] has been approved by the Central Joint V.A.D. Committee, by which it is hoped that training ma[illegible] be given to members after demobilisation in th[illegible] different branches of health and welfare work. W[illegible] shall look forward to receiving further particulars o[illegible] this scheme.

---

Among the barbarities perpetrated by German[illegible] on their prisoners none strikes our imaginatio[illegible] more relentlessly than the performance by thei[illegible] doctors of operations on the British without anæs[illegible]thetics. An Army Chaplain, writing this month i[illegible] the *Nineteenth Century* of incidents which he ha[illegible] taken pains to verify, tells his readers that th[illegible] hospitals were in many cases places of torture rathe[illegible] than of healing. "It was no uncommon thing fo[illegible] a man to be on the operating table for an hour at [illegible] stretch without an anæsthetic." The men ha[illegible] invariably spoken with the utmost gratitude of th[illegible] doctors who treated them humanely, and we lea[illegible] "there are about half-a-dozen German Army su[illegible]geons whose names will ever be honoured by th[illegible] prisoners who passed through their hands." Bu[illegible] for the most part it must be said the surgeons pu[illegible]sued a deliberate policy of either neglecting th[illegible] British wounded or operating with an evil spirit o[illegible] malice which overpowered all decent profession[illegible] instincts. Can we wonder that nurses traine[illegible] under such men did not do conspicuously well whe[illegible] put to the test?

---

The King held an Investiture on December 1[illegible] the usual Saturday ceremony having been postpone[illegible] for two weeks during His Majesty's absence abroa[illegible] Many distinguished persons, including seven re[illegible]cipients of the Victoria Cross, were personall[illegible] decorated by the King, who conferred the Orde[illegible] of the Royal Red Cross 1st Class on Assistan[illegible] Matron Margaret Weir, T.F.N.S., and on Matro[illegible] Emily Power, B.R.C.S. His Majesty conferre[illegible] the Royal Red Cross 2nd Class on Sister Anni[illegible] Buyers and Sister Beatrice Thomas, Q.A.I.M.N.S. on Sister Mary Collier, Sister Anna Stuart, an[illegible] Staff Nurse Matilda Tate, Q.A.I.M.N.S.R.; o[illegible]

**ROUND THE HOSPITALS—(*continued*).**

Matron Elizabeth Price and Matron Evelyn Pugh, C.N.S.; on Mrs. Minnie Scott, V.A.D.; on Assistant Matron Alvira Stevens and Sister Letitia Kelly, American N.S. The King also conferred the Military Medal on Miss Mary Campbell and Miss Gertrude Johnston, V.A D.; and on Sister Meta Hodge Canadian A.N.S. After the Investiture the members of the Military and Civil Nursing Services had the honour of being invited to a reception at Marlborough House, where they were presented to Queen Alexandra.

---

THE commemoration service celebrated by the British community on December 9 at the grave of the late Edith Cavell in the Tir National Cemetery in Brussels was exceedingly impressive. The service was conducted by the brave Chaplain, the Rev. H. S. T. Gahan, who so ably shepherded his little flock throughout the whole period of the German occupation, and was privileged to minister to Edith Cavell in her last hours. The British Minister, Sir F. H. Villiers, attended the memorial service, and laid upon the grave, which has been identified from the German plan of the cemetery, a wreath from King George and Queen Mary in memory of "a brave and splendid Englishwoman." Another wreath, bound also with the British colours, was sent in the name of the British Legation and Colony, and a third was contributed by the American Minister, Mr. Brand Whitlock. In a steady downpour, to the accompaniment of a high gale, the ceremony was brought to a solemn close by the graveside where a large and distinguished company stood reverently to do honour to Nurse Cavell. The King and Queen of the Belgians laid their tribute —a wreath inscribed "To Edith Cavell—Elizabeth" —personally on the grave shortly after they entered Brussels.

---

WE have seldom read a sadder story than that revealed at the inquest on the body of Mary E. E. M. Boshell, daughter of a Colombia merchant, who died at the Nursing Home of St. John and St. Elizabeth, St. John's Wood, on December 6. The Coroner stated that Miss Boshell had been engaged in nursing in Belgium, that she fell ill and was nursed in one of the hospitals in France, that she was brought home and stayed at the house of a friend until she was taken to the Nursing Home. Under her pillow was found a box containing drugs. Her brother, Lieutenant Boshell, said in his evidence that his sister was exceptionally healthy and strong. She had a desire "to do something with her life" and came to England from South America in 1916. She became engaged to a member of the French Diplomatic Service, who was called to the Colours on the eve of their marriage, and was killed directly after. She then enrolled as a V.A.D. nurse in Belgium and served in proximity to the battle-fields of Belgium for a year. At one time the town in which she worked was under shell and she behaved with gallantry. After this there was a history of sleeplessness; a French doctor gave her veronal, and directed this treatment should be continued. Evidence given by Dr. Jewesbury established the fact that numerous injections of morphia had been made on the right fore-arm, and three hypodermic syringes with tubes of solution of morphia were found among her possessions. The inquiry was adjourned.

---

A PLEASANT "interview" and account of Princess Mary's tour in France is given by Grace Curnoch in the *Daily Mail* of the 6th inst. The most notable memento of her visit brought back was a little gold identity disc presented to her prior to her departure by Miss Rachel Crowdy, R.R.C., on behalf of the V.A.D.s, who assembled in large numbers to do honour to Princess Mary. Among the other gifts presented to the Princess is a little brass box bearing the Corps Badge, presented to her by the Field Ambulance Nursing Yeomanry, made of the bases of two small shells. Another box made from the bases of two shells, which gave immense pleasure, was presented to the Princess on the Boulogne boat as she started on her return journey. It bore an inscription to H.R.H. Princess Mary from the Nursing V.A.D.s in France, November 20-30, 1918," and came on board in a fish basket as a "surprise present." Among other gifts were a beautiful little ivory case containing scent presented by the English people working in Rouen; a well-modelled bronze statuette of a *poilu;* a small ivory statuette of Joan of Arc; another of a Boulogne fisherwoman; and a silver souvenir spoon. The Princess was profoundly impressed by the energy and self-sacrifice shown by the great army of British girls working behind the lines, and more particularly with the labours of the ambulance and motor drivers, whose work, done in all weathers, under all conditions, and frequently by night as well as by day, seemed to H.R.H. far beyond all praise.

---

THE Local Government Board has made an astonishing blunder in laying down the necessary qualifications for the tuberculosis health visitor. We learn that "she should, whenever practicable, have had at least three months' training at a tuberculosis dispensary, and also training in social work . . . evidence of further experience in health visiting, and a knowledge of sanitary work will also be an advantage." In other words (as the *Times* puts it), if she does not know anything about tuberculosis or sanitary work it will not matter very much. Health visiting will never become a strong reconstructive influence, never do more than just satisfy the conscience of the Town Council, while the training of these women is left to chance. At present it seems to be in a similar stage to that of hospital matrons fifty years ago, when any worthy lady recommended by the chairman's wife could secure the post. Meantime we have Dr. Niven, Medical Officer for Health in Manchester, outlining the virtues that "must be possessed" by health visitors, and talking about the absolute need for tact, patience, energy, sympathy, and cheerfulness,

**ROUND THE HOSPITALS—(*continued*).**

with "intelligent and tried advice." The list of duties and of the subjects on which she must offer expert advice point to long and specialised training.

Neither the Local Government Board nor the medical officers of health seem to realise that the qualities absolutely necessary for the health visitor are exactly the qualities which hospital training tends to develop in women, and that no other form of training can evolve these qualities with anything like the same success. It is most deplorable that owing to narrowness in the curriculum hospital nurses are not available in greater numbers for the important work of health visiting. We believe that the future success of tuberculosis and health visiting depends entirely on securing the women who have been trained with a view to this branch of work under the best auspices in hospitals, and have subsequently taken specialised courses to fit them for sanitary inspection.

The Basil Blackwood Day Nursery at 16 Cornwall Gardens, S.W., for the accommodation of widowed officers' little children in cases where their mothers are engaged all day, is proving a great success, and points to the need for a further extension of the crèche system among the middle classes which we have more than once urged. But widows with families, it appears, not only need a nursery for the little ones while they themselves are at work, but are also put to great straits in finding suitable lodgings. Accordingly the day nursery, which was founded as a memorial to Lord Basil Blackwood, of the Grenadier Guards, is to be supplemented by a hostel for mothers and babies next door, organised "by mothers for mothers." It will in the end be self-supporting, but the sum of £1,500 is needed to start it. We cannot doubt this small capital will speedily be forthcoming in response to Lady Victoria Plunkett's appeal.

At the Horton Infirmary, Banbury, the salaries have been raised so that sisters receive £45 to £50, staff nurses £40, probationers £15, £18, £20. The hospital serves a wide district, and since the installation of the $x$-rays apparatus has largely increased its value to the surrounding neighbourhood. A private ward has been added, which has proved very attractive, and expansion in this direction is demanded. An increase in the nursing staff has led to the erection of temporary nurses' quarters in a bungalow in the grounds, which, though comfortably furnished and fitted with electric light, is merely a makeshift until a proper Nurses' Home can be constructed. The managers are anxious to secure a complete reconstruction or large extension of the hospital as the Banbury and district war memorial, and there is a very good case for the adoption of this course. It is believed that even after the demobilisation a large proportion of Army men will need recurrent treatment at the hands of the civil hospital authorities. We earnestly hope that if Banbury people adopt this scheme they will remember the great part played by nurses in the war and take steps to ensure that the new Nurses' Home is in all respects, in beauty and convenience, an adequate memorial to the sisters who have given their lives for the country.

A nurse of unusual distinction, Mrs. Teresa Eden Richardson, Lady of Grace of the Order of St. John of Jerusalem, has lately died at Bath. She nursed the wounded in four wars, and holds the South African Medal, the Order of the Crown of Japan, the Japanese Red Cross Order of Merit, two Greek medals, and the Mons Star. Her experiences as a Red Cross nurse in Japan during the Russian War were unique, and are recounted in a clever book which she afterwards published, "In Japanese Hospitals in War Time." Mrs. Richardson was sixty-eight in 1914, but she went at once to Belgium to give her services, and worked in one of the Antwerp hospitals throughout the bombardment. She was one of the last to escape from the town, and she never quite recovered from the privations endured at that time. A good woman filled with the spirit of a true vocation.

Any who are inclined to complain at the continuance of food regulations now that the seas are open once more will find their grumbles die away in dismay if they consider the larger outlook on the world's food supplies this winter. Mr. Hoover, the American Food Administrator, has recently declared that the spectre of famine abroad now haunts the abundance of our table at home. There are conditions of famine in Europe that will be beyond our powers to remedy. There are 40,000,000 people in Russia to whom but little access to food can be obtained this winter. Their transportation is demoralised to complete anarchy, and shortly many of their ports will be frozen. Mr. Hoover frankly exposes his dread of "starvation beyond all human power to allay." A powerful organisation is being formed to organise the Continental food supplies, for Germany has sucked food and animals from all those masses of people she has dominated, and left behind her total wreckage of social institutions. Never in the history of civilisation have more potent influences been set to work to rescue the starving. Is it not a very little thing which is asked of us to continue awhile still to tread the path of strict economy in food?

☛ It is our invariable practice to pay for all items of news which we may publish. The minimum payment is 5s. Paragraphs of about twenty lines in length—that is to say, of 150 words or less—are preferred. Contributions should be addressed to The Editor, The Hospital, 28 and 29 Southampton Street, Strand, London, W.C. 2, and, to be in time for the current week's issue, should reach him by the first post on Mondays.

